# KDM4A, involved in the inflammatory and oxidative stress caused by traumatic brain injury-hemorrhagic shock, partly through the regulation of the microglia M1 polarization

**DOI:** 10.1186/s12868-023-00784-6

**Published:** 2023-03-03

**Authors:** Jimin Cai, Yang Yang, Jiahui Han, Yu Gao, Xin Li, Xin Ge

**Affiliations:** 1 Department of Critical Care Medicine, Wuxi 9th People’s Hospital Affiliated to Soochow University, 214000 Wuxi, Jiangsu P.R. China; 2 Department of Neurosurgery, Central Hospital of Jinzhou, 121001 Jinzhou, Liaoning P.R. China; 3 Department of Anesthesiology, Wuxi 9th People’s Hospital Affiliated to Soochow University, 214000 Wuxi, Jiangsu P.R. China; 4 Orthopedic Institution of Wuxi City, 214000 Wuxi, Jiangsu P.R. China

**Keywords:** KDM4A, TBI, HS, Microglia, M1 polarization

## Abstract

**Background:**

Microglial polarization and the subsequent neuroinflammatory response and oxidative stress are contributing factors for traumatic brain injury (TBI) plus hemorrhagic shock (HS) induced brain injury. In the present work, we have explored whether Lysine (K)-specific demethylase 4 A (KDM4A) modulates microglia M1 polarization in the TBI and HS mice.

**Results:**

Male C57BL/6J mice were used to investigate the microglia polarization in the TBI + HS model in vivo. Lipopolysaccharide (LPS)-induced BV2 cells were used to examine the mechanism of KDM4A in regulating microglia polarization in vitro. We found that TBI + HS resulted in neuronal loss and microglia M1 polarization in vivo, reflected by the increased level of Iba1, tumor necrosis factor (TNF)-α, interleukin (IL)-1β, malondialdehyde (MDA) and the decreased level of reduced glutathione (GSH). Additionally, KDM4A was upregulated in response to TBI + HS and microglia were among the cell types showing the increased level of KDM4A. Similar to the results in vivo, KDM4A also highly expressed in LPS-induced BV2 cells. LPS-induced BV2 cells exhibited enhanced microglia M1 polarization, and enhanced level of pro-inflammatory cytokines, oxidative stress and reactive oxygen species (ROS), while this enhancement was abolished by the suppression of KDM4A.

**Conclusion:**

Accordingly, our findings indicated that KDM4A was upregulated in response to TBI + HS and microglia were among the cell types showing the increased level of KDM4A. The important role of KDM4A in TBI + HS-induced inflammatory response and oxidative stress was at least partially realized through regulating microglia M1 polarization.

**Supplementary Information:**

The online version contains supplementary material available at 10.1186/s12868-023-00784-6.

## Background

Traumatic brain injury (TBI) refers to changes in brain function or other evidence of brain pathology caused by external forces. Despite some advances in treatment, TBI remains a leading cause of death and morbidity [1]. TBI has a complex pathogenesis, and the degree of brain damage depends not only on the primary degree, but also on the severity of secondary damage caused by hypoxia, hypotension, and other injuries [2, 3]. Given the associated mechanisms of injury, the occurrence of TBI is often accompanied by hemorrhagic shock (HS) [4]. Studies have shown that HS can double the morbidity and mortality associated with TBI [5, 6]. After TBI, HS caused by massive blood loss is also seemed as the important risk factor for systemic inflammation, barrier disruption and multiple organ failure leading to death [7–9]. In case of HS, brain could increase the rate of cerebral oxygen extraction and cardiac output from internal organs to protected body from shock-induced hypoxia and reperfusion injury. At the beginning, these changes could protect brain, while, the ischemia in other organs and the secretion of proinflammatory mediators will leads to the secondary injury [10]. When massive blood loss or shock persists for too long without adequate resuscitation, these compensatory mechanisms begin to fail, leading to cellular metabolic disorders, inflammatory cell activation, and neuronal cell death in the brain, which seriously affects the prognosis of patients [11]. In particular, inflammation and oxidative stress seemed as the most common and important cellular events following TBI + HS [12]. Therefore, it is of great interest to investigate the underlying mechanisms of injury that occurred in TBI and HS.

Lysine (K)-specific demethylase 4 A (KDM4A, also known as JMJD2A, JHDM3A, and KIA0677) is a member of the Jumonji domain 2 (JMJD2) families [13, 14]. Through KDM4A activity, H3K9me3 demethylation promotes an open chromatin state, contributing to the transcription activation of promoter regions [15, 16]. At present, researches on KDM4A mainly focus on transcriptional regulation, which can either stimulate or inhibit gene transcription. Study showed that KDM4A could regulate the expression of pro-inflammatory factors and participate in the occurrence of diabetic vascular complications [17]. Besides that, KDM4A inhibition has been proved represses neuroinflammation and improves functional recovery in ischemic stroke [18]. It was also found that the expression of KDM4A was significantly increased in lipopolysaccharide (LPS)-induced microglia [19], and downregulation of KDM4A could inhibit M1 polarization of macrophages [20]. Microglia as the principal resident macrophages of the brain and spinal cord comprise 5–12% of brain cells and act as primary effector cells. These cells play an important role in the brain’s innate immunity, neuronal homeostasis, and neuro inflammatory pathologies [21, 22]. Microglia could be rapidly activated by infection, inflammation or brain injury, and the M1-like phenotype of microglia has been shown to be associated with neuroinflammation in neurodegenerative diseases [23]. However, the function of KDM4A in microglial M1 polarization in TBI caused HS has not been addressed.

Accordingly, in this work, we implied that KDM4A may play a key role in microglia M1 polarization in the TBI + HS induced brain injury.

## Results

### TBI + HS accelerated microglia M1 polarization and aggravated the microglia-mediated inflammatory response and oxidative stress

**Fig. 1:**
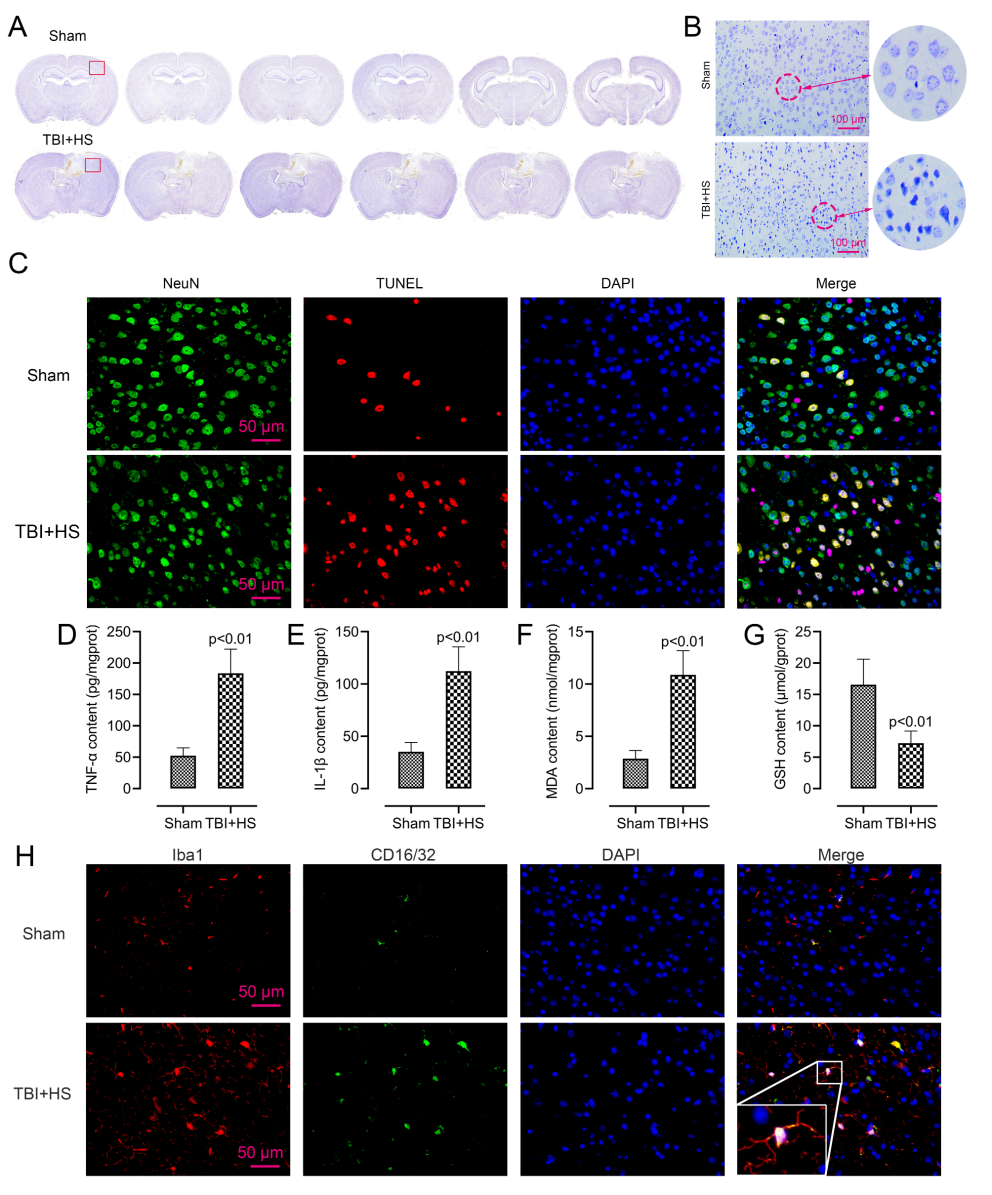
TBI + HS accelerated microglia M1 polarization and aggravated the microglia-mediated inflammatory response and oxidative stress. (A). The lesions in the brain sections of the sham and TBI + HS mice was measured by Nissl-staining. (B). Enlargement Nissl staining images of neuronal loss in the ischemic penumbra area (the red box area in A) of sham and TBI + HS mice. (C). The images of double immunofluorescent staining for NeuN (green) and TUNEL (red) in sham and TBI + HS mice. (D-G). The content of TNF-α, IL-1β, MDA and GSH in sham and TBI + HS mice were measured by detection kits (n = 6). Unpaired t-test was used to analyze data. (H). The images of double immunofluorescent staining for CD16/32 (green) and Iba1 (red) in sham and TBI + HS mice. Blue fluorescence represented DAPI. Data were collected from three independent experiments and expressed as mean ± SD.

At first, we observed the neurons in the cerebral cortex surrounding the injury site. The images of complete mice brain sections with Nissl-staining at different Bregma levels were provided as the Fig. 1A. Compared with the sham, TBI + HS mice exhibited obviously lesions in images. After that, the images were enlarged to explore the neuronal damage in ischemic penumbra area of mice. As shown in Fig. 1B, the neurons were clear and intact in the sham mice. By contrast, damaged neurons displayed irregular cell bodies, shrinkage, and hyperchromatic nuclei in significantly greater numbers in TBI + HS mice. Neuron-specific marker NeuN (green) and TUNEL (red) double immunofluorescent staining was used to detect the injury of neurons after TBI + HS. As in Fig. 1C, compared with the sham, an enhanced number of TUNEL positive cells were shown in TBI + HS mice. This result was similar with our Nissl-staining images, showing that TBI + HS caused neurons damage in the cerebral cortex of mice.

Considering that TBI and HS often led to biochemical, cellular, and physiological changes like inflammation and generation of oxidative stress, we measured the content of tumor necrosis factor (TNF)-α, interleukin (IL)-1β, malondialdehyde (MDA) and reduced glutathione (GSH) in different groups of mice by corresponding detection kits. As shown in Fig. 1D and E, TBI + HS mice have a higher content of TNF-α and IL-1β than the sham mice, indicating that TBI + HS promotes the expression of pro-inflammatory factors. Similarly, the results in Fig. 1F and G indicated that TBI + HS mice have a relatively higher content of MDA and lower content of GSH, which means that TBI + HS exacerbated the oxidative stress in brain injured mice.

Besides that, microglia could become rapidly activated in response to brain injury, to specifically evaluate the effect of TBI + HS on the M1 polarization state, a proinflammatory phenotype of microglia, CD16/32 was co-labeled with the microglia marker Iba1 in the cerebral cortex tissues of mice, respectively. Compared with the sham, an enhanced level of Iba1 (red) was shown in TBI + HS mice, which means the activation of microglia. CD16/32 (green) as the marker of M1 type microglia also showed an increased level after TBI + HS (Fig. 1H). The merged images showed that the number of CD16/32 and Iba1 double-positive cells was increased and displayed a clear co-localization in the ischemic penumbra area of mice after TBI + HS. This finding indicated that TBI + HS accelerated microglia M1 polarization in mice.

### KDM4A was upregulated in the activated microglia of TBI + HS mice

**Fig. 2:**
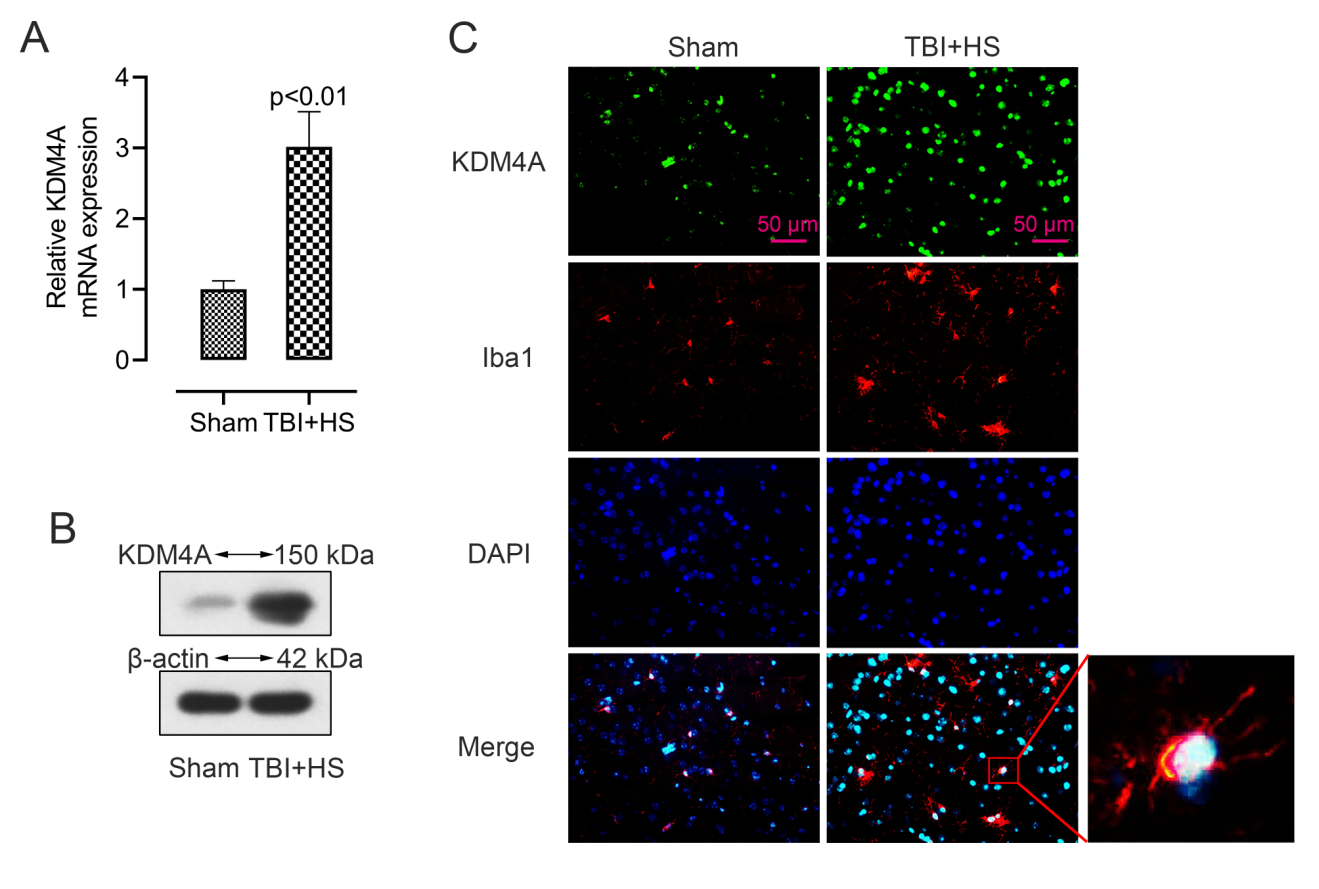
KDM4A was upregulated in the activated microglia of TBI + HS mice. (A-B). The mRNA and protein level of KDM4A in the mice cerebral cortex tissues were accessed by qRT-PCR and western blot (n = 6). Unpaired t-test was used to analyze data. (C). The images of double immunofluorescent staining for KDM4A (green) and Iba1 (red) in sham and TBI + HS mice. Blue fluorescence represented DAPI. Data were collected from three independent experiments and expressed as mean ± SD. Full-length blots are presented in Supplementary Figure S1

Then, we measured the expression level of KDM4A in brain tissues. As shown in Fig. 2A and B, both mRNA and protein levels of KDM4A were significantly increased in TBI + HS mice as compared to the sham one. Since KDM4A has been reported to be upregulated in microglia [19], we further investigated the cellular localization and expression of KDM4A in the cerebral cortex tissues after TBI + HS, using double immunofluorescent staining of KDM4A and microglial marker Iba1. As demonstrated in Fig. 2C, the green light of KDM4A was greatly enhanced after TBI + HS, and the red light of Iba1 was also increased. The merged images showed that KDM4A was upregulated in the activated microglia in the TBI + HS mice. This finding indicated that KDM4A was upregulated in response to TBI + HS and microglia were among the cell types showing the increased level of KDM4A.

### KDM4A was upregulated in LPS-induced BV2 cells

Previous studies have linked LPS-induced activation of microglia and the production of pro-inflammatory factors and oxidative stress to brain damage and neuron degeneration [24, 25]. When BV2 cells are stimulated by LPS, microglia served as tissue-resident macrophages in the brain, and release M1 phenotype pro-inflammatory cytokines. Thus, we used LPS-induced BV2 cells to explore the potential regulatory mechanism of KMD4A on TBI + HS caused inflammation and oxidative stress in vitro.

**Fig. 3:**
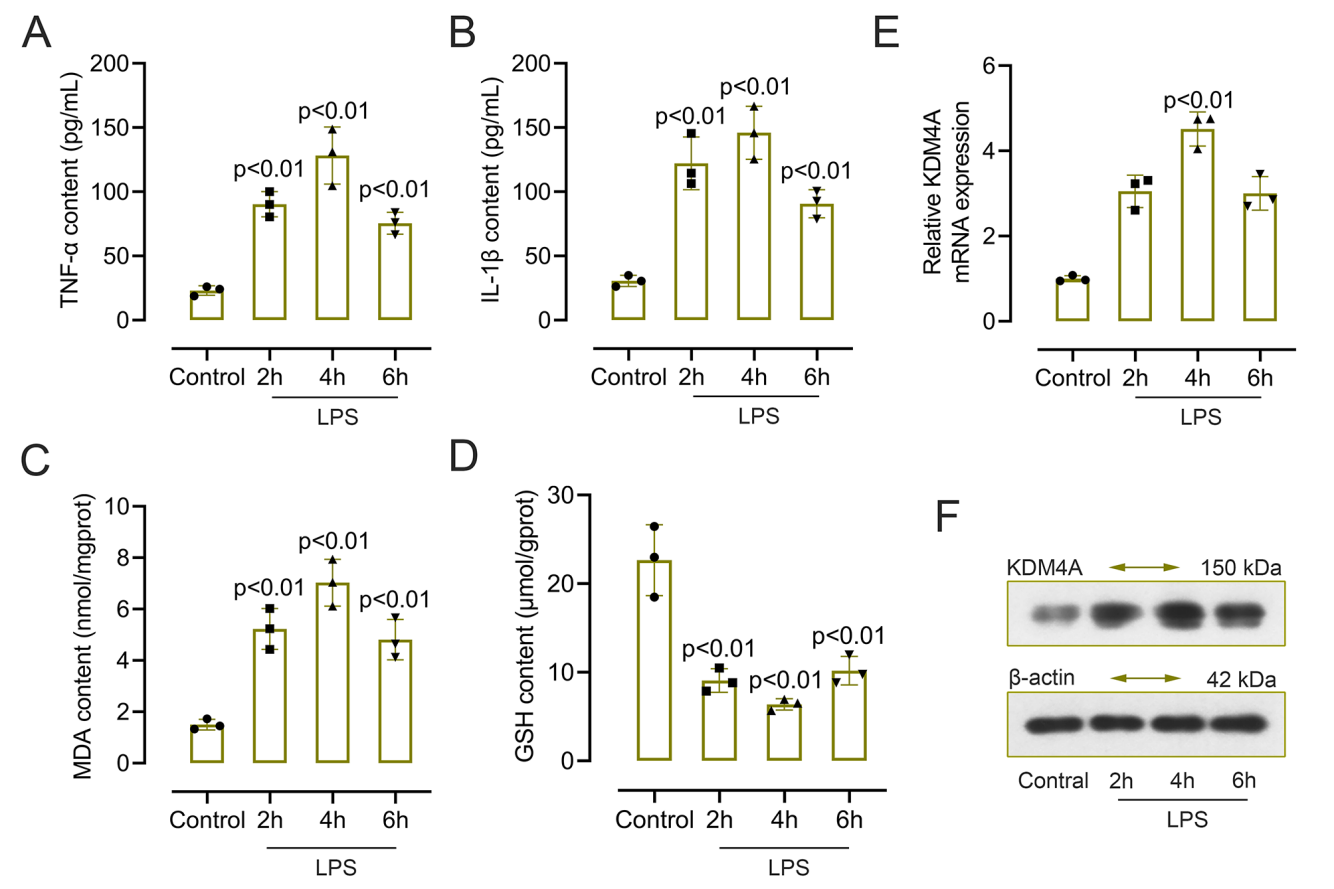
KDM4A was upregulated in LPS-induced BV2 cells. (A-D). The content of TNF-α, IL-1β, MDA and GSH in LPS-induced BV2 cells was measured by detection kits (n = 3). (E-F). The mRNA and protein level of KDM4A in LPS-induced BV2 cells was accessed by qRT-PCR and western blot (n = 3). One-way analysis of variance (ANOVA) and Dunnett’s multiple comparisons test were used to analyze data. Data were collected from three independent experiments and expressed as mean ± SD. Full-length blots are presented in Supplementary Figure S2

To investigate the pro-inflammatory responses in LPS-induced BV2 cells, we measured the content of TNF-α ad IL-1β in Fig. 3A and B. We found that the levels of proinflammatory cytokines were many-fold upregulated in LPS-induced BV2 cells, especially in the cells incubated for 4 h. Furthermore, the content of MDA and GSH showed a different result in LPS-induced cells, with increased content of MDA and a decreased content of GSH (Fig. 3C and D). Then, we analyzed the levels of KDM4A mRNA and protein in the LPS-induced BV2 cells. As shown in Fig. 3E and F, both KDM4A mRNA and protein levels were significantly increased in LPS-induced BV2 cells when compared with the control. Collectively, these results were the same as the results in vivo, and 4 h was selected as the optimized LPS stimulation time for further experiment.

### KDM4A involved in the inflammation and oxidative stress in LPS-induced BV2 cells

**Fig. 4:**
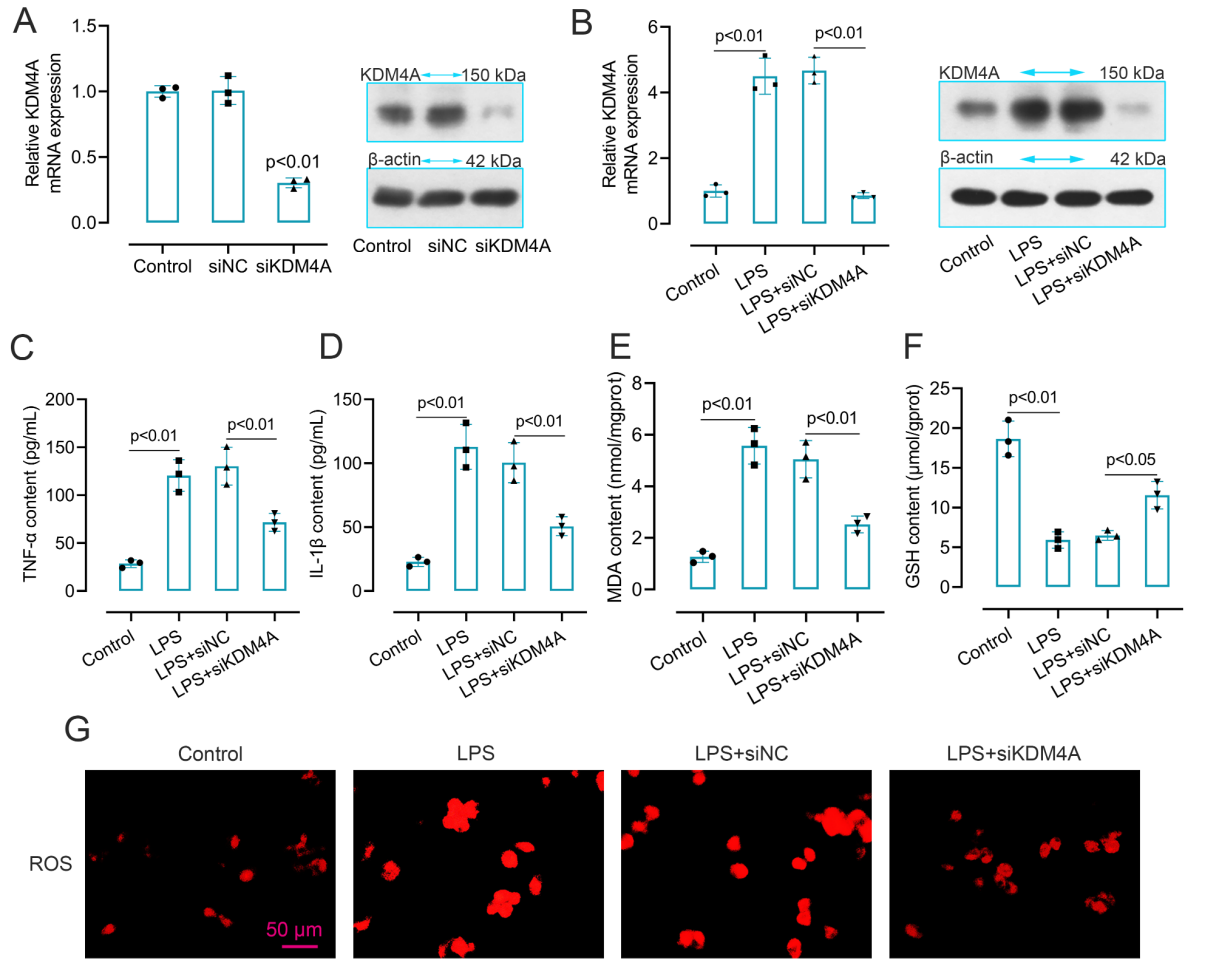
KDM4A involved in the inflammation and oxidative stress in LPS-induced BV2 cells. (A). The mRNA and protein expression levels of KDM4A in KDM4A down-regulated BV2 cells (n = 3). (B). The mRNA and protein expression levels of KDM4A in KDM4A down-regulated LPS-induced BV2 cells (n = 3). (C-F). The content of TNF-α, IL-1β, MDA and GSH in KDM4A inhibited LPS-induced BV2 cells were measured by detection kits (n = 3). ANOVA and Tukey’s multiple comparisons test were used to analyze data. (G). ROS level in KDM4A down-regulated LPS-induced BV2 cells. Data were collected from three independent experiments and expressed as mean ± SD. Full-length blots are presented in Supplementary Figure S3

To further determine the role of KDM4A in TBI, KDM4A was knockdown in BV2 cells, and the efficiency was confirmed by qRT-PCR and western blot in Fig. 4A. Based on this, we knockdown KDM4A in LPS-induced BV2 cells and the efficiency was confirmed in Fig. 4B. Then, we measured the content of TNF-α, IL-1β, MDA and GSH. As shown in Fig. 4C-F, LPS-induced inflammatory and oxidative stress reactions were both counteracted by the suppression of KDM4A. The level of reactive oxygen species (ROS) in LPS-induced BV2 cells was tested in Fig. 4G. Consistent with MDA, the level of ROS was aggravated in LPS-induced BV2 cells, while the suppression of KDM4A abolished this enhancement. These findings reflected that KDM4A involved in the inflammation and oxidative stress in LPS-induced BV2 cells.

### Suppressed KDM4A abolished microglia M1 polarization in LPS-induced BV2 cells

**Fig. 5:**
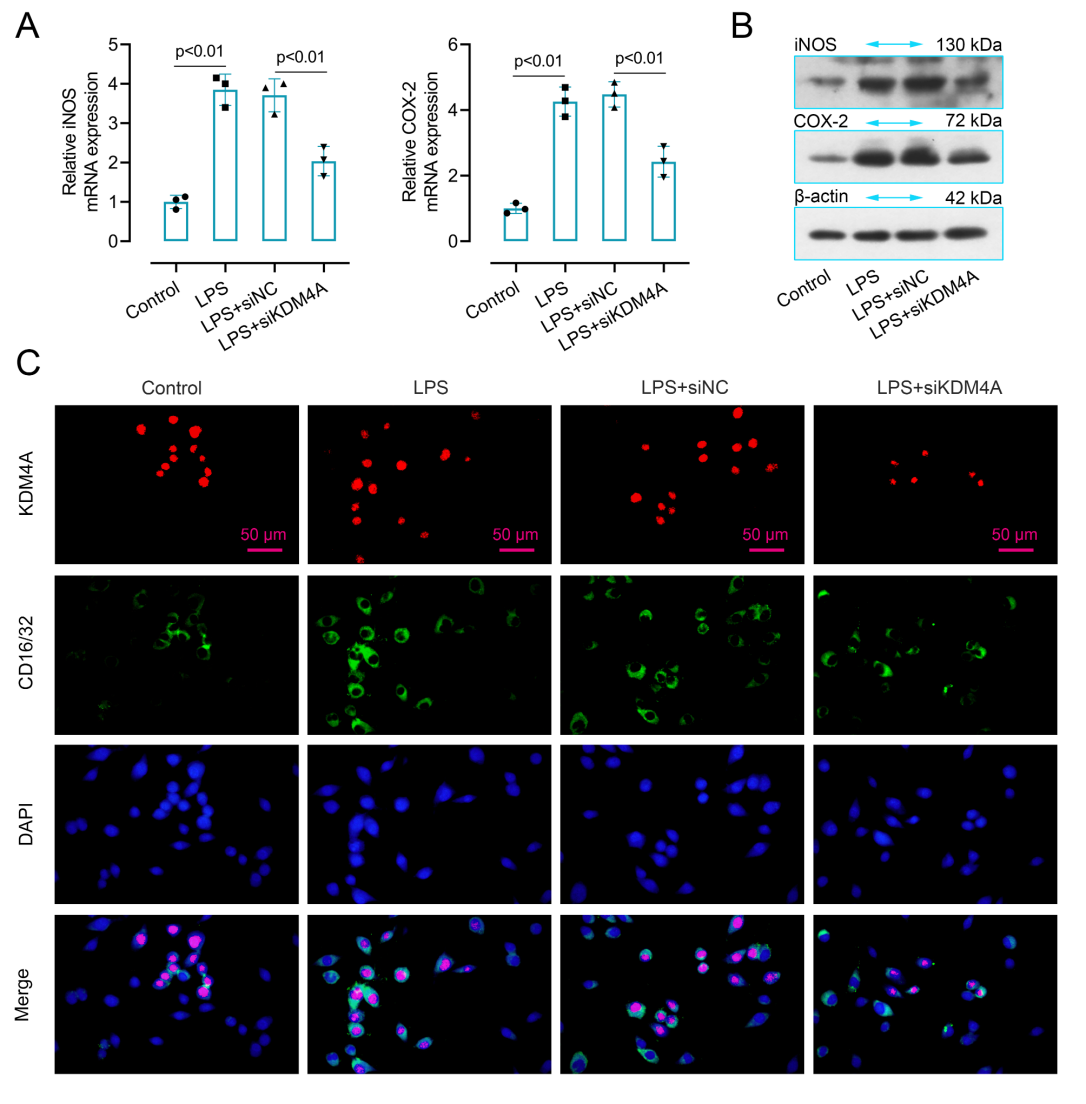
Suppressed KDM4A abolished microglia M1 polarization in LPS-induced BV2 cells. (A-B). The mRNA and protein expression levels of iNOS and COX-2 in KDM4A down-regulated BV2 cells (n = 3). ANOVA and Tukey’s multiple comparisons test were used to analyze data. (C). Images of double immunofluorescent staining for KDM4A (red) and CD16/32 (green) in KDM4A inhibited LPS-induced BV2 cells. Blue fluorescence represented DAPI. Data were collected from three independent experiments and expressed as mean ± SD. Full-length blots are presented in Supplementary Figure S4

To confirm the involvement of KDM4A in M1 microglia polarization of LPS-induced BV2 cells, we further analyzed the expression level of inducible nitric oxide synthase (iNOS), cyclooxygenase-2 (COX-2) and CD16/32, the markers of microglia M1 polarization. As shown in Fig. 5A and B, both mRNA and protein levels of iNOS and COX-2 were significantly increased in LPS-induced BV2 cells as compared to the control, while on the contrary, suppressed KDM4A alleviated the enhanced level of iNOS and COX-2 in cells. The double immunofluorescence images of KDM4A and CD16/32 in Fig. 5C also drew the same conclusion. The images showed that LPS stimulation enhanced the expression level of CD16/32 and KDM4A in BV2 cells. However, this enhanced trend was abolished by KDM4A knockdown, reflected by the decreased level of CD16/32 and KDM4A.

## Discussion

**Fig. 6:**
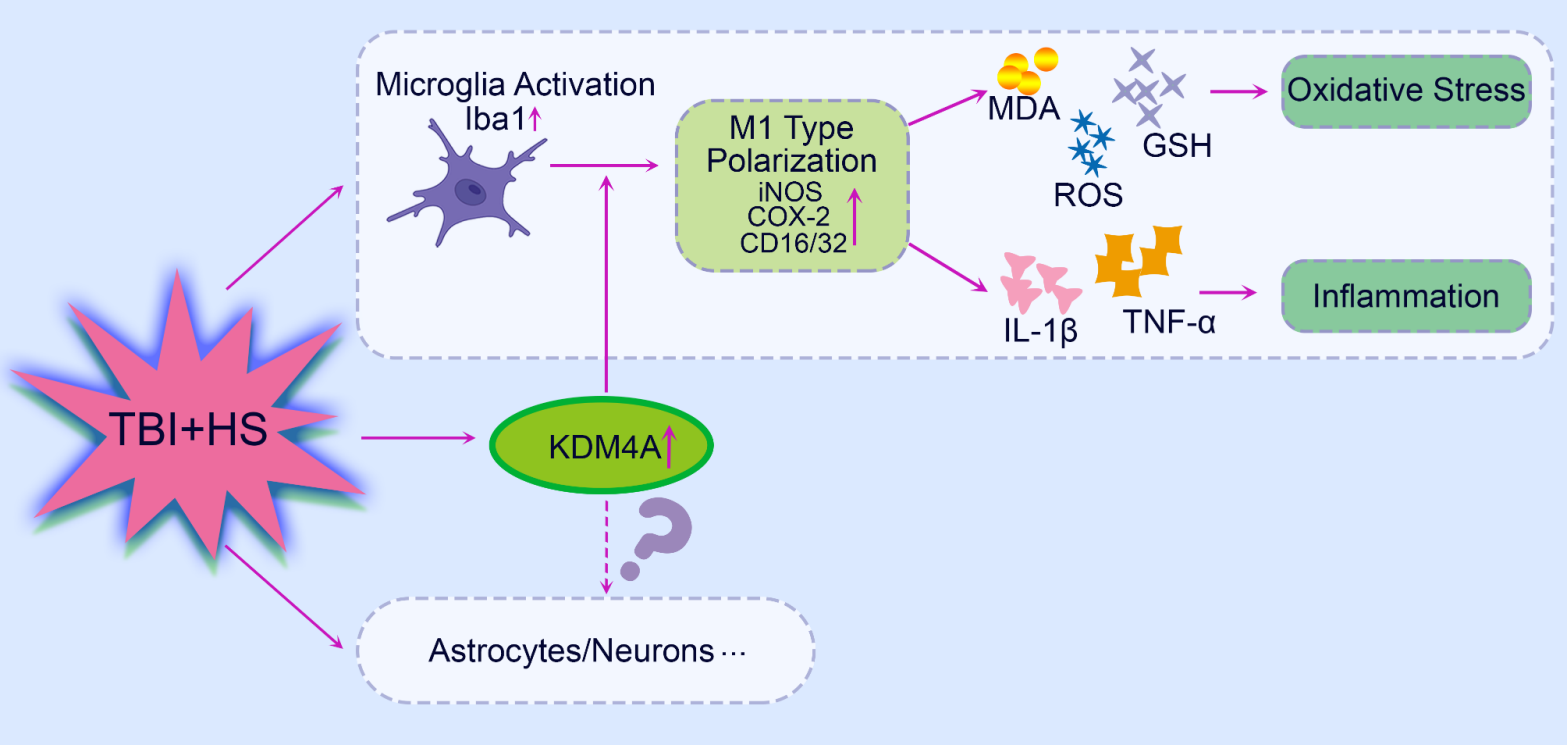
The simple scheme indicated that TBI + HS resulted in enhanced microglia M1 polarization. KDM4A was upregulated in response to TBI + HS and microglia were among the cell types showing the increased level of KDM4A. Inhibition of KDM4A alleviated the inflammation and oxidative stress, which was related with microglia activation. Accordingly, our findings indicated that the important role of KDM4A in TBI + HS induced inflammatory response and oxidative stress was at least partially realized through microglia M1 polarization

TBI and HS are often concomitantly occurred in clinical patients because of the multiple injuries [26]. The patients are faced with worse outcomes and increased morbidity when TBI occurs in conjunction with HS [27–29]. In traumatic settings, when HS and TBI are found in combination, the likelihood of early death is approximately 80% [30]. Therefore, we explored the role of KDM4A in the TBI + HS combined model induced brain injury. In TBI + HS caused brain injury, inflammation and oxidative stress are considered to be the most important pathbiological features [12, 31]. In the present study, we found that TBI + HS resulted in enhanced microglia M1 polarization. KDM4A was upregulated in response to TBI + HS and microglia were among the cell types showing the increased level of KDM4A. Inhibition of KDM4A alleviated the inflammation and oxidative stress, which was related with microglia activation. Accordingly, our findings indicated that the enhanced level of KDM4A was involved in the TBI + HS induced inflammatory response and oxidative stress. Our results show that KDM4A plays an important role in TBI + HS-induced brain injury, which is at least partly achieved by activation of the microglia (Fig. 6).

In this work, we used male mice to construct a TBI + HS model in vivo. Studies showed that, after TBI, more severe impairments was found in males than in females [32, 33], which may due to the protective effect of female sex hormones, estrogen, and progesterone [34, 35]. More importantly, in this work, we mainly explored the function role of KDM4A in TBI + HS caused microglia activation. A study reported that after brain injury, male mice have a faster and more robust microglia activation and peripheral macrophage recruitment compared to females [36, 37]. Therefore, based on these findings, we chose male mice as the subject for in vivo study. The Nissl staining results proved that, compared with the sham, TBI + HS aggravated the neuronal loss in mice. We further measured the expression level of inflammatory and oxidative stress related factors in TBI + HS mice. Studies proved that the massive production of free radicals can cause lipid peroxidation, protein degradation and genotoxicity, resulting in cell and tissue damage [6]. Additionally, the enhanced antioxidant response and decreased inflammation have been proved could alleviate the brain damage [38–40]. Similar to the previous researches, TBI + HS mice displayed enhanced level of TNF-α, IL-1β and MDA, inhibited level of GSH. This finding indicated that TBI + HS caused an enhanced inflammatory response and oxidative stress in mice. After TBI, microglia was quickly activated and released various inflammatory mediators, including TNF-α, IL-1B, IL6, NO and ROS, which have been proved to be related in some neurodegenerative diseases [41, 42]. Based on this, we measured the expression level of Iba1 and CD16/32, the marker of microglia M1 polarization, in the TBI + HS mice. The images reflected that the percentage of CD16/32 and Iba1 double-positive M1-like cells was significantly increased in the cerebral cortex tissues of mice after TBI + HS. These results confirmed that microglia were activated after TBI + HS.

Since KDM4A has been reported to be upregulated in microglia [19]. Also, microglia as the most important resident immune cells in the central nervous system plays an important role in TBI caused injury [43, 44]. Therefore, we examined the expression of KDM4A in microglia by double immunofluorescent staining and the result showed that KDM4A was enhanced in activated microglia. In order to explore the potential relationship of KDM4A and microglia activation in vitro, LPS-induced BV2 cells was used in this work for further exploration. We found that the level of pro-inflammatory factors was many-fold upregulated in LPS-induced BV2 cells. Similarly, a study also showed that LPS could induce significant increases in the level of TNF-α, IL-6, and MCP-1 in mice [45]. Studies have confirmed that free radicals were elevated after TBI and HS, which could cause lipid peroxidation [46, 47]. In our research, the content of MDA and GSH showed an opposite result in LPS-induced cells. As an index of lipid peroxidation, MDA level increase, whereas there is a decrease in the anti-oxidant GSH. However, silenced KDM4A showed a completely different trends in cells, a decreased level of proinflammatory factors and MDA, an enhanced level of GSH. Besides that, the accumulation of ROS could change the expression of apoptosis genes and inflammatory mediators from low expression to high expression, thereby inducing apoptosis [48]. In our results, LPS-induced cells exhibited a high level of ROS, while, KDM4A inhibition decreased the level of ROS. To confirm the involvement of KDM4A in M1 microglia polarization, we further analyzed the expression level of the markers of M1 polarization. Enzymes such as COX-2 and iNOS are reported to be involved in excessive production of proinflammatory mediators [51]. We found that both mRNA and protein levels of iNOS and COX-2 were significantly increased in LPS-induced BV2 cells, while on the contrary, suppressed KDM4A alleviated the enhanced level. LPS stimulation enhanced the green fluorescence of CD16/32 in LPS-induced BV2 cells. However, the enhanced fluorescence intensity was abolished by KDM4A knockdown. Therefore, these results may imply that the LPS-activated M1 microglia polarization was seriously blunted by repression KDM4A in BV2 cells.

In addition, our study also found that there was a certain basal expression of KDM4A in the brain tissue of the sham mice, suggesting that KDM4A may also play a role in maintaining normal brain function. Besides that, it is worth noticed that the expression of KDM4A was not only increased in microglia in the brain tissue of TBI + HS mice, but also seemed to be abnormally increased in other cells. Studies reported that the other cells in the brain, such as astrocytes and neurons, also play an important role in TBI + HS induced brain injury [49, 50]. KDM4A has been shown to be related with the differentiation of neural stem cells into astrocytes [51]. Therefore, we cannot rule out that KDM4A may play a more important role in TBI + HS by affecting these cells, which is considered as the limitation of this study. In future work, we will further investigate whether KDM4A participates in TBI + HS induced brain injury by affecting other cells.

In summary, in this work, we found that KDM4A played an important role in TBI + HS mice. KDM4A was upregulated in response to TBI + HS and microglia were among the cell types showing the increased level of KDM4A. Activated microglia has been proved exerts neurotoxic effects by producing and releasing ROS, inflammatory cytokines, which consequently triggers oxidative stress and severe inflammation [52]. Accordingly, we suspected that KDM4A was involved in TBI + HS induced inflammatory response and oxidative stress via affecting microglia M1 polarization. However, the specific mechanism still needs more study to confirm. And this will also be an important point in our further study.

### Conclusion

In summary, this work demonstrated that KDM4A was upregulated in response to TBI + HS and microglia were among the cell types showing the increased level of KDM4A. Further studies confirmed that the role of KDM4A in TBI + HS induced inflammatory response and oxidative stress was partly realized by regulating the M1 polarization of microglia.

### Methods

### Mice TBI and HS model

Male C57BL/6J mice (12–15 weeks old) used in this study were randomly divided into two groups, sham (n = 18) and TBI + HS (n = 18). Mice were maintained on 12 h light/dark cycle with temperature of 22℃, with accessed food and water for a week.

The mice model of combined TBI plus HS was produced by a mild-to-moderate controlled cortical impact, followed by a pressure controlled HS [53–56]. Inguinal cut-down and insertion of double-side femoral arterial catheters were accomplished under conditions following the anesthesia. After placement of the mouse in a stereotaxic frame, a 5-mm craniotomy was performed over the left parietotemporal cortex with a cranial drill, and the bone flap was removed. Then, a 3-mm flat-tip impactor was deployed at a velocity of 5 m/sec and a depth of 1 mm. After that, the wound was quickly cleaned to control bleeding, dental cement was used to fill the wound, and the skin of the head was sutured. After TBI, mice remained a target mean arterial blood pressure (MAP) of 25–27 mm Hg (HS phase) for a total of 35 min. After completion of the HS phase, a 90-min prehospital phase was initiated and 20 mL/kg of lactated Ringer’s (LR) was rapidly infused. Additional aliquots of 10 mL/kg were infused as needed to achieve a MAP of 70 mm Hg. After the hospital phase, intubation was removed, vascular was ligated, and mice were disinfected and sutured before being returned to their cages. The mice were sacrificed 24 h after recovery, and the cerebral cortex tissue around the injury was taken for subsequent experimental detection.

### Nissl staining

Paraffin-embedded tissues were cut into 5 μm thick sections and sequentially stained with xylene (1330-20-7, Aladdin, China), anhydrous ethanol (10,009,218, Sinopharm Chemical Reagent Co., Ltd, China), 95%, 85%, and 75% alcohol, and distilled water. The sections were then stained with 0.5% cresol purple (71,044,080, Sinopharm Chemical Reagent Co., Ltd, China) for 10 min. Subsequently, the sections were rinsed with distilled water and differentiated with 0.25% glacial acetic acid ethanol solution (FX11610, Tianjin Kemiou Chemical Reagent, China). Next, the sections were washed with anhydrous ethanol twice (with 5 min for each time), followed by washing with xylene twice (with 10 min for each time). Finally, the sections were observed and captured by a microscope.

### Enzyme-linked immunosorbent assay (ELISA) and other detection kits

Sample tissues were weighted and diluted with nine-fold normal saline, followed by mechanical homogenization for 10 min. The protein concentration was measured by a BCA Protein Assay Kit (P0011, Beyotime, China). Cells were collected and centrifuged for further detection. The content of TNF-α and IL-1β were separately tested by the mouse TNF-a ELISA kit (EK282, MULTI SCIENCES, China) and mouse IL-1β ELISA kit (EK201B, MULTI SCIENCES, China) according to the manufacture’s instruction. The content of malondialdehyde (MDA) and reduced glutathione (GSH) were separately tested by the MDA assay kit (A003-1, Nanjing Jiancheng Biotech, China) and Reduced GSH assay kit (A006-2, Nanjing Jiancheng Biotech, China) according to the manufacture’s instruction.

### Immunofluorescence staining

Paraffin-embedded tissues were cut into 5 μm thick sections and sequentially stained with xylene (Aladdin, China), 95%, 85%, and 75% alcohol, and distilled water. The brain sections with antigen retrieval were treated with 1% BSA (A602440-0050, Sangon Biotech, China) for 15 min at room temperature and then incubated with primary antibodies against CD16/32 (1:50, Proteintech Cat# 65057-1-Ig, RRID: AB_2918365, China), Iba1 (1:100, Abcam Cat# ab178847, RRID: AB_2832244 USA), KDM4A (1:200, 6E10G4, NOVUS, USA) overnight at 4℃. The slides were incubated with corresponding secondary antibodies (1:200), FITC labeled goat anti mouse (Abcam Cat# ab6785, RRID: AB_955241, UK) or Cy3 labeled goat anti rabbit (Thermo Fisher Scientific Cat# A27039, RRID: AB_2536100, USA) IgG for 90 min. Then, the slides were washed in phosphate buffered saline and counterstained with 4, 6-diamidino-2-phenylindole (DAPI, D106471, Aladdin, China). Cells were fixed with 4% paraformaldehyde (80,096,618, Sinopharm Chemical Reagent Co., Ltd, China) solution for 15 min at room temperature. Cell permeabilization was achieved by administration of 0.1% Triton X-100 (ST795, Beyotime, China) solution for 30 min. Subsequently, cover slips were blocked with a 1% BSA solution for 15 min and then incubated with anti CD16/32 antibody overnight at 4℃. Then cover slips were incubated with a FITC labeled goat anti mouse antibody for 1 h and mounted with DAPI solution. Fluorescence was captured on a DP73 microscope system (BX53, OLYMPUS, Japan).

### Cell culture and transfection

Mouse microglia cells BV2 (RRID: CVCL_0182, iCELL, China) was cultured in DMEM medium (G4510, Service Biotech, China) supplemented with 10% fetal calf serum (11,011 − 8611, Tianhang Biotech, China) at 37℃ with 5% CO_2_ atmosphere. For preparation, lipofectamine 3000 (L3000015, Invitrogen, USA) was used to achieve KDM4A inhibition according to the manufacture’s instruction. In some section, cells were treated with 10 ng/mL LPS (L8880, Solarbio, China) for further investigation.

### Quantitative real time PCR (qRT-PCR)

TRIpure (RP1001, BioTeke, China) was adopted to extract total RNA from tissues and cells and RNA purity was assessed using spectrophotometer (Nano 2000, Thermo, USA). BeyoRT II M-MLV reverse transcriptase (D7160L, Beyotime, China) used in this part to synthesis cDNA. The SYBR GREEN (SY1020, Solarbio, China) and 2×Taq PCR MasterMix (PC1150, Solarbio, China) reagents were employed for qPCR analysis and the results were analyzed by an ExicyclerTM 96 fluorescence quantitative instrument (BIONEER, Korea). β-actin was the internal control. The 2^−ΔΔCT^ method was adopted to quantify the results. The primer sequences were as follows: KDM4A forward: 5’ CGCCAATAGCGACAAGTA 3’, and reverse: 5’ TGCCGAAGTAAAGGTAGGG 3’; COX-2 forward: 5’ AAAACCTCGTCCAGATGCTA 3’, and reverse: 3’ TTGAGGAGAACAGATGGGAT 5’; iNOS forward: 5’ TTGGAGCGAGTTGTGGATTG 3’, and reverse: 5’ GTGAGGGCTTGGCTGAGTGA 3’.

### Western blot analysis

Tissues or cells were cultured in lysis buffer (P0013, Beyotime, China) with phenylmethanesulfonyl fluoride (PMSF, ST506, Beyotime, China) for 5 min. Then the lysates were centrifuged for 5 min at 4℃ and the protein concentration was detected by using the BCA protein assay kit (P0011, Beyotime, China). After that, the proteins were electrophoresed in sodium dodecyl sulfate polyacrylamide gel electrophoresis (SDS-PAGE, P0015, Beyotime, China) for separation and transferred onto the polyvinylidene fluoride membrane (IPVH00010, Millipore, China). The membranes were blocked with 5% skim milk powder (Yili, China) and then incubated with primary antibodies against KDM4A (ABclonal Cat# A7953, RRID: AB_2770069, China), iNOS (ABclonal Cat# A0312, RRID: AB_2757120, China), COX-2 (ABclonal Cat# A3560, RRID: AB_2922972, China), or β-actin (Santa Cruz Biotechnology Cat# sc-47,778, RRID: AB_626632, USA) at 4℃ overnight. After washing, the membrane was cultured with horseradish peroxidase-conjugated (HRP) goat anti rabbit antibody (1:5000, Beyotime Cat# A0208, RRID: AB_2892644, China) at room temperature for 45 min. Finally, the signals were estimated by enhanced chemiluminescence reagent (ECL, P0018, Beyotime, China) as suggested. The results were analyzed by Gel-Pro-Analyzer.

### ROS detection

ROS was measured by ROS detection kit (BB-47,051, Bestbio, China) and the procedures were according to manufacturer’s instructions. The neurons were incubated with ROS probe for 30 min at 37℃. Following three washes with PBS for 5 min, the cells were photographed under a BX53 fluorescence microscope (OLYMPUS, Japan).

### Statistical analysis

All statistical analyses were conducted via GraphPad Prism software (GraphPad, Inc., La Jolla, CA, USA). The data were collected from three independent experiments and indicated as the mean ± standard deviation (SD). Normality was evaluated by Shapiro-Wilk’s test and the homogeneity of variances was tested by F test or Brown-Forsythe test. For comparison between two groups, unpaired t test with or without Welch’s correction was employed according to the homoscedasticity of variances. For comparisons among multiple groups, one-way analysis of variance (ANOVA) was applied for the data with normality and homogeneity, Kruskal-Wallis’s test was applied for non-normality. P value less than 0.05 was accepted as statistically significant.

## Electronic supplementary material

Below is the link to the electronic supplementary material.

Additional file 1

## Data Availability

The data presented in this study are available on request from the corresponding author.

## References

[1] Dong T, Zhi L, Bhayana B, Wu MX (2016). Cortisol-induced immune suppression by a blockade of lymphocyte egress in traumatic brain injury. J Neuroinflamm.

[2] Rhind SG, Crnko NT, Baker AJ, Morrison LJ, Shek PN, Scarpelini S, Rizoli SB (2010). Prehospital resuscitation with hypertonic saline-dextran modulates inflammatory, coagulation and endothelial activation marker profiles in severe traumatic brain injured patients. J Neuroinflamm.

[3] Chesnut RM, Marshall LF, Klauber MR, Blunt BA, Baldwin N, Eisenberg HM, Jane JA, Marmarou A, Foulkes MA (1993). The role of secondary brain injury in determining outcome from severe head injury. J trauma.

[4] Nikolian VC, Dekker SE, Bambakidis T, Higgins GA, Dennahy IS, Georgoff PE, Williams AM, Andjelkovic AV, Alam HB (2018). Improvement of blood-brain Barrier Integrity in Traumatic Brain Injury and hemorrhagic shock following treatment with Valproic Acid and Fresh Frozen plasma. Crit Care Med.

[5] McMahon CG, Yates DW, Campbell FM, Hollis S, Woodford M (1999). Unexpected contribution of moderate traumatic brain injury to death after major trauma. J trauma.

[6] Wald SL, Shackford SR, Fenwick J (1993). The effect of secondary insults on mortality and long-term disability after severe head injury in a rural region without a trauma system. J trauma.

[7] Huber-Lang M, Lambris JD, Ward PA (2018). Innate immune responses to trauma. Nat Immunol.

[8] Dutton RP (2012). Haemostatic resuscitation. Br J Anaesth.

[9] Halbgebauer R, Braun CK, Denk S, Mayer B, Cinelli P, Radermacher P, Wanner GA, Simmen HP, Gebhard F, Rittirsch D (2018). Hemorrhagic shock drives glycocalyx, barrier and organ dysfunction early after polytrauma. J Crit Care.

[10] Deniz T, Agalar C, Agalar F, Comu FM, Caglayan O, Alpay Y, Saygun O (2010). The effect of hypothermia on splanchnic flows and lung in a two-hit hemorrhagic shock model. J Surg Res.

[11] Rao G, Xie J, Hedrick A, Awasthi V (2015). Hemorrhagic shock-induced cerebral bioenergetic imbalance is corrected by pharmacologic treatment with EF24 in a rat model. Neuropharmacology.

[12] Simon DW, McGeachy MJ, Bayır H, Clark RS, Loane DJ, Kochanek PM (2017). The far-reaching scope of neuroinflammation after traumatic brain injury. Nat reviews Neurol.

[13] Couture JF, Collazo E, Ortiz-Tello PA, Brunzelle JS, Trievel RC (2007). Specificity and mechanism of JMJD2A, a trimethyllysine-specific histone demethylase. Nat Struct Mol Biol.

[14] Ng SS, Kavanagh KL, McDonough MA, Butler D, Pilka ES, Lienard BM, Bray JE, Savitsky P, Gileadi O, von Delft F (2007). Crystal structures of histone demethylase JMJD2A reveal basis for substrate specificity. Nature.

[15] Guerra-Calderas L, González-Barrios R, Herrera LA, Cantú de León D, Soto-Reyes E (2015). The role of the histone demethylase KDM4A in cancer. Cancer Genet.

[16] Loh YH, Zhang W, Chen X, George J, Ng HH (2007). Jmjd1a and Jmjd2c histone H3 lys 9 demethylases regulate self-renewal in embryonic stem cells. Genes Dev.

[17] Qi H, Jing Z, Xiaolin W, Changwu X, Xiaorong H, Jian Y, Jing C, Hong J. Histone Demethylase JMJD2A Inhibition Attenuates Neointimal Hyperplasia in the Carotid Arteries of Balloon-Injured Diabetic Rats via Transcriptional Silencing: Inflammatory Gene Expression in Vascular Smooth Muscle Cells. Cellular physiology and biochemistry: international journal of experimental cellular physiology, biochemistry, and pharmacology 2015, 37(2):719–734. 10.1159/000430390 26356263

[18] Liu Y, Zhao L, Zhang J, Lv L, Han K, Huang C, Xu Z (2021). Histone demethylase KDM4A inhibition represses neuroinflammation and improves functional recovery in ischemic stroke. Curr Pharm Design.

[19] Das A, Kim SH, Arifuzzaman S, Yoon T, Chai JC, Lee YS, Park KS, Jung KH, Chai YG (2016). Transcriptome sequencing reveals that LPS-triggered transcriptional responses in established microglia BV2 cell lines are poorly representative of primary microglia. J Neuroinflamm.

[20] Wang X, Wang S, Yao G, Yu D, Chen K, Tong Q, Ye L, Wu C, Sun Y, Li H, Oncotarget et al. 2017, 8(70):114442–114456. 10.18632/oncotarget.17748 PMC5777704 29383092

[21] Graeber MB, Streit WJ (2010). Microglia: biology and pathology. Acta Neuropathol.

[22] Lawson LJ, Perry VH, Dri P, Gordon S (1990). Heterogeneity in the distribution and morphology of microglia in the normal adult mouse brain. Neuroscience.

[23] Loane DJ, Kumar A. Microglia in the TBI brain: The good, the bad, and the dysregulated. Experimental neurology 2016, 275 Pt3(0 3):316–327. 10.1016/j.expneurol.2015.08.018 PMC4689601 26342753

[24] Han JH, Lee YS, Im JH, Ham YW, Lee HP, Han SB, Hong JT. Astaxanthin Ameliorates Lipopolysaccharide-Induced Neuroinflammation, Oxidative Stress and Memory Dysfunction through Inactivation of the Signal Transducer and Activator of Transcription 3 Pathway. Marine drugs 2019, 17(2). 10.3390/md17020123 PMC6410230 30781690

[25] Fleisher-Berkovich S, Abramovitch-Dahan C, Ben-Shabat S, Apte R, Beit-Yannai E (2009). Inhibitory effect of carnosine and N-acetyl carnosine on LPS-induced microglial oxidative stress and inflammation. Peptides.

[26] Qi L, Cui X, Dong W, Barrera R, Coppa GF, Wang P, Wu R (2014). Ghrelin protects rats against traumatic brain injury and hemorrhagic shock through upregulation of UCP2. Ann Surg.

[27] Muller CR, Courelli V, Lucas A, Williams AT, Li JB, Dos Santos F, Cuddington CT, Moses SR, Palmer AF, Kistler EB (2021). Resuscitation from hemorrhagic shock after traumatic brain injury with polymerized hemoglobin. Sci Rep.

[28] Mayer AR, Dodd AB, Rannou-Latella JG, Stephenson DD, Dodd RJ, Ling JM, Mehos CJ, Robertson-Benta CR, Pabbathi Reddy S, Kinsler RE (2021). 17α-Ethinyl estradiol-3-sulfate increases survival and hemodynamic functioning in a large animal model of combined traumatic brain injury and hemorrhagic shock: a randomized control trial. Crit Care (London England).

[29] Leung LY, Deng-Bryant Y, Shear D, Tortella F (2016). Experimental models combining TBI, hemorrhagic shock, and Hypoxemia. Methods in molecular biology. (Clifton NJ).

[30] Williams AM, Bhatti UF, Dennahy IS, Graham NJ, Nikolian VC, Chtraklin K, Chang P, Zhou J, Biesterveld BE, Eliason J (2019). Traumatic brain injury may worsen clinical outcomes after prolonged partial resuscitative endovascular balloon occlusion of the aorta in severe hemorrhagic shock model. J trauma acute care Surg.

[31] Cornelius C, Crupi R, Calabrese V, Graziano A, Milone P, Pennisi G, Radak Z, Calabrese EJ, Cuzzocrea S (2013). Traumatic brain injury: oxidative stress and neuroprotection. Antioxid Redox Signal.

[32] Mollayeva T, Mollayeva S, Colantonio A (2018). Traumatic brain injury: sex, gender and intersecting vulnerabilities. Nat reviews Neurol.

[33] Späni CB, Braun DJ, Van Eldik LJ (2018). Sex-related responses after traumatic brain injury: considerations for preclinical modeling. Front Neuroendocr.

[34] Engler-Chiurazzi EB, Brown CM, Povroznik JM, Simpkins JW (2017). Estrogens as neuroprotectants: estrogenic actions in the context of cognitive aging and brain injury. Prog Neurobiol.

[35] Stein DG (2015). Embracing failure: what the Phase III progesterone studies can teach about TBI clinical trials. Brain Injury.

[36] Villapol S, Loane DJ, Burns MP (2017). Sexual dimorphism in the inflammatory response to traumatic brain injury. Glia.

[37] Eyolfson E, Carr T, Khan A, Wright DK, Mychasiuk R, Lohman AW (2020). Repetitive mild traumatic brain injuries in mice during adolescence cause sexually dimorphic behavioral deficits and Neuroinflammatory Dynamics. J Neurotrauma.

[38] Fang J, Wang H, Zhou J, Dai W, Zhu Y, Zhou Y, Wang X, Zhou M. Baicalin provides neuroprotection in traumatic brain injury mice model through Akt/Nrf2 pathway. Drug design, development and therapy 2018, 12:2497–2508. 10.2147/DDDT.S163951 PMC6089097 30127597

[39] Wang J, Jiang C, Zhang K, Lan X, Chen X, Zang W, Wang Z, Guan F, Zhu C, Yang X (2019). Melatonin receptor activation provides cerebral protection after traumatic brain injury by mitigating oxidative stress and inflammation via the Nrf2 signaling pathway. Free Radic Biol Med.

[40] Zeng J, Chen Y, Ding R, Feng L, Fu Z, Yang S, Deng X, Xie Z, Zheng S (2017). Isoliquiritigenin alleviates early brain injury after experimental intracerebral hemorrhage via suppressing ROS- and/or NF-κB-mediated NLRP3 inflammasome activation by promoting Nrf2 antioxidant pathway. J Neuroinflamm.

[41] Streit WJ, Mrak RE, Griffin WS (2004). Microglia and neuroinflammation: a pathological perspective. J Neuroinflamm.

[42] More SV, Kumar H, Kim IS, Koppulla S, Kim BW, Choi DK (2013). Strategic selection of neuroinflammatory models in Parkinson’s disease: evidence from experimental studies. CNS Neurol Disord Drug Target.

[43] Harry GJ (2012). Neuroinflammation: a need to understand microglia as resident cells of the developing brain. Neurotoxicology.

[44] Li Y, Yang YY, Ren JL, Xu F, Chen FM, Li A (2017). Exosomes secreted by stem cells from human exfoliated deciduous teeth contribute to functional recovery after traumatic brain injury by shifting microglia M1/M2 polarization in rats. Stem Cell Res Ther.

[45] Fernández-Calle R, Vicente-Rodríguez M, Gramage E, Pita J, Pérez-García C, Ferrer-Alcón M, Uribarri M, Ramos MP, Herradón G (2017). Pleiotrophin regulates microglia-mediated neuroinflammation. J Neuroinflamm.

[46] Cristofori L, Tavazzi B, Gambin R, Vagnozzi R, Vivenza C, Amorini AM, Di Pierro D, Fazzina G, Lazzarino G (2001). Early onset of lipid peroxidation after human traumatic brain injury: a fatal limitation for the free radical scavenger pharmacological therapy?. J Invest medicine: official publication Am Federation Clin Res.

[47] Eroğlu O, Deniz T, Kisa Ü, Atasoy P, Aydinuraz K (2017). Effect of hypothermia on apoptosis in traumatic brain injury and hemorrhagic shock model. Injury.

[48] Simon HU, Haj-Yehia A, Levi-Schaffer F (2000). Role of reactive oxygen species (ROS) in apoptosis induction. Apoptosis: an international journal on programmed cell death.

[49] Karve IP, Taylor JM, Crack PJ (2016). The contribution of astrocytes and microglia to traumatic brain injury. Br J Pharmacol.

[50] Burda JE, Bernstein AM, Sofroniew MV. Astrocyte roles in traumatic brain injury. Experimental neurology 2016, 275 Pt3(0 3):305–315. 10.1016/j.expneurol.2015.03.020 PMC4586307 25828533

[51] Cascante A, Klum S, Biswas M, Antolin-Fontes B, Barnabé-Heider F, Hermanson O (2014). Gene-specific methylation control of H3K9 and H3K36 on neurotrophic BDNF versus astroglial GFAP genes by KDM4A/C regulates neural stem cell differentiation. J Mol Biol.

[52] Baek JY, Jeong JY, Kim KI, Won SY, Chung YC, Nam JH, Cho EJ, Ahn TB, Bok E, Shin WH et al. Inhibition of Microglia-Derived Oxidative Stress by Ciliary Neurotrophic Factor Protects Dopamine Neurons In Vivo from MPP^+^ Neurotoxicity. International journal of molecular sciences 2018, 19(11). 10.3390/ijms19113543 PMC6274815 30423807

[53] Hemerka JN, Wu X, Dixon CE, Garman RH, Exo JL, Shellington DK, Blasiole B, Vagni VA, Janesko-Feldman K, Xu M (2012). Severe brief pressure-controlled hemorrhagic shock after traumatic brain injury exacerbates functional deficits and long-term neuropathological damage in mice. J Neurotrauma.

[54] Kumar A, Henry RJ, Stoica BA, Loane DJ, Abulwerdi G, Bhat SA, Faden AI (2019). Neutral sphingomyelinase inhibition alleviates LPS-Induced Microglia activation and neuroinflammation after experimental traumatic brain Injury. J Pharmacol Exp Ther.

[55] Shein SL, Shellington DK, Exo JL, Jackson TC, Wisniewski SR, Jackson EK, Vagni VA, Bayır H, Clark RS, Dixon CE (2014). Hemorrhagic shock shifts the serum cytokine profile from pro- to anti-inflammatory after experimental traumatic brain injury in mice. J Neurotrauma.

[56] Xie Y, Xu M, Deng M, Li Z, Wang P, Ren S, Guo Y, Ma X, Fan J, Billiar TR (2019). Activation of pregnane X receptor sensitizes mice to Hemorrhagic Shock-Induced Liver Injury. Hepatology (Baltimore MD).

