# Supplementary material for: KDM4A, involved in the inflammatory and oxidative stress caused by traumatic brain injury-hemorrhagic shock, partly through the regulation of the microglia M1 polarization
Source: BMC Neurosci. 2023 Mar 3;24:17. doi: 10.1186/s12868-023-00784-6 (PMC9983262; doi:10.1186/s12868-023-00784-6)

Supplementary material

for

**KDM4A, involved in the inflammatory and oxidative stress caused by traumatic brain injury-hemorrhagic shock, partly through the regulation of the microglia M1 polarization**

Jimin Cai^1, †^, Yang Yang^2, †^, Jiahui Han^1^, Yu Gao^1^ , Xin Li^3, *^, Xin Ge^1, 4, *^

^1^Department of ICU, Wuxi 9th People’s Hospital Affiliated to Soochow University, Wuxi, Jiangsu 214000, P.R. China.

^2^Department of Neurosurgery, Central Hospital of Jinzhou, Jinzhou, Liaoning 121001, P.R. China.

^3^Department of Anesthesiology, Wuxi 9th People’s Hospital Affiliated to Soochow University, Wuxi, Jiangsu 214000, P.R. China.

^4^Orthopedic Institution of Wuxi City, Wuxi, Jiangsu 214000, P.R. China.

†These authors contributed equally.

*Correspondence: Dr. Xin Ge, Department of ICU, Wuxi 9th People’s Hospital Affiliated to Soochow University,Wuxi, Jiangsu 214000, P.R. China. Orthopedic Institution of Wuxi City, Wuxi, Jiangsu 214000, P.R. China.

*Co-Correspondence: Mr. Xin Li, Department of Anesthesiology, Wuxi 9th People’s Hospital Affiliated to Soochow University,Wuxi, Jiangsu 214000, P.R. China.

Tel/Fax: +86-510-85867999

Email address: gexin2021@suda.edu.cn


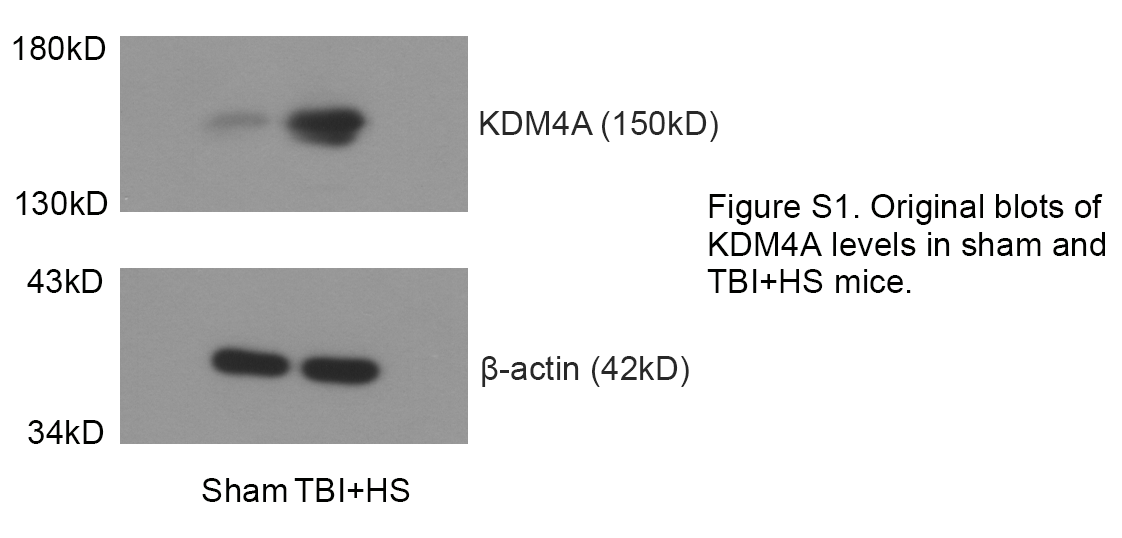


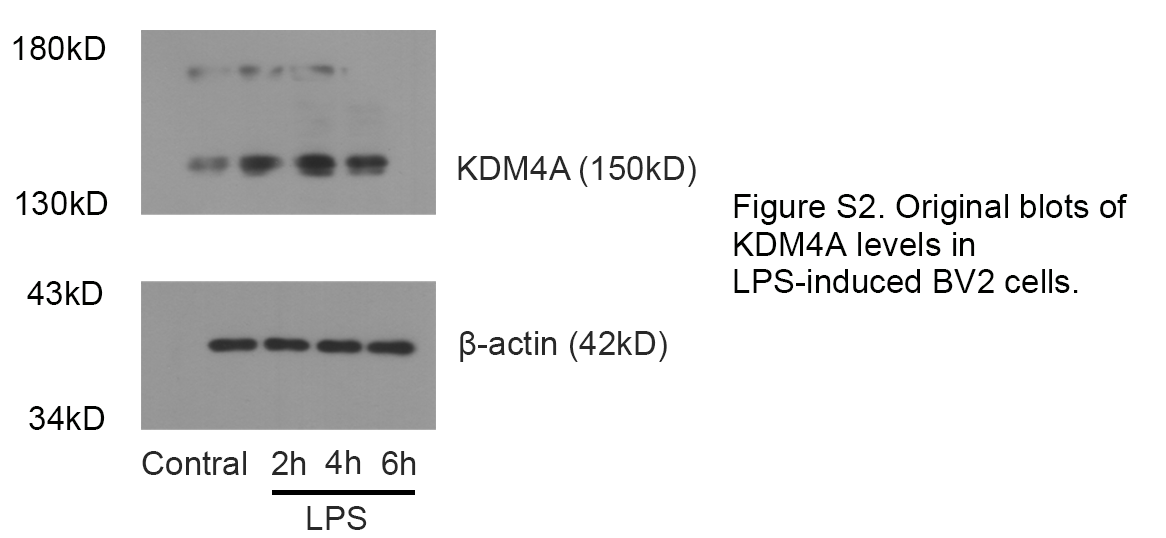


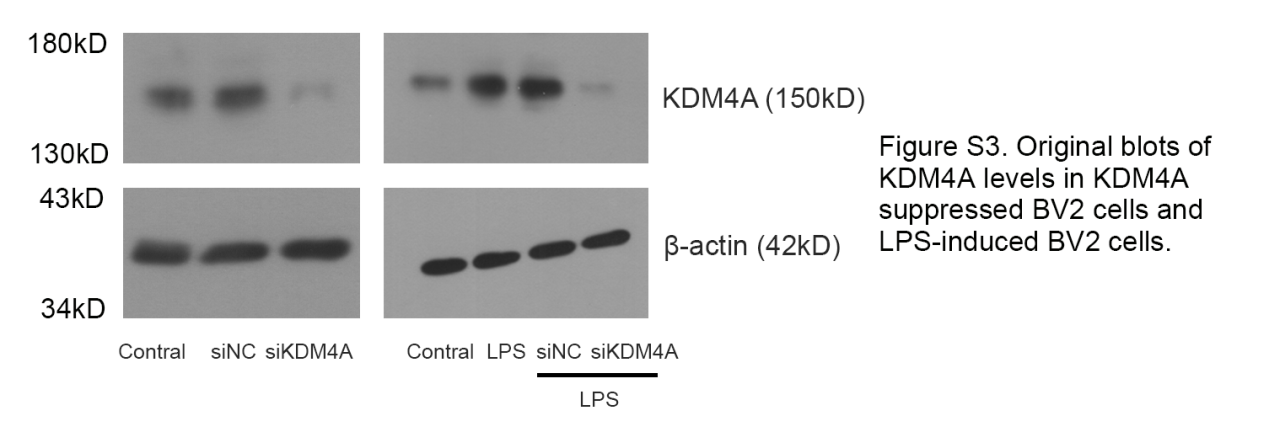


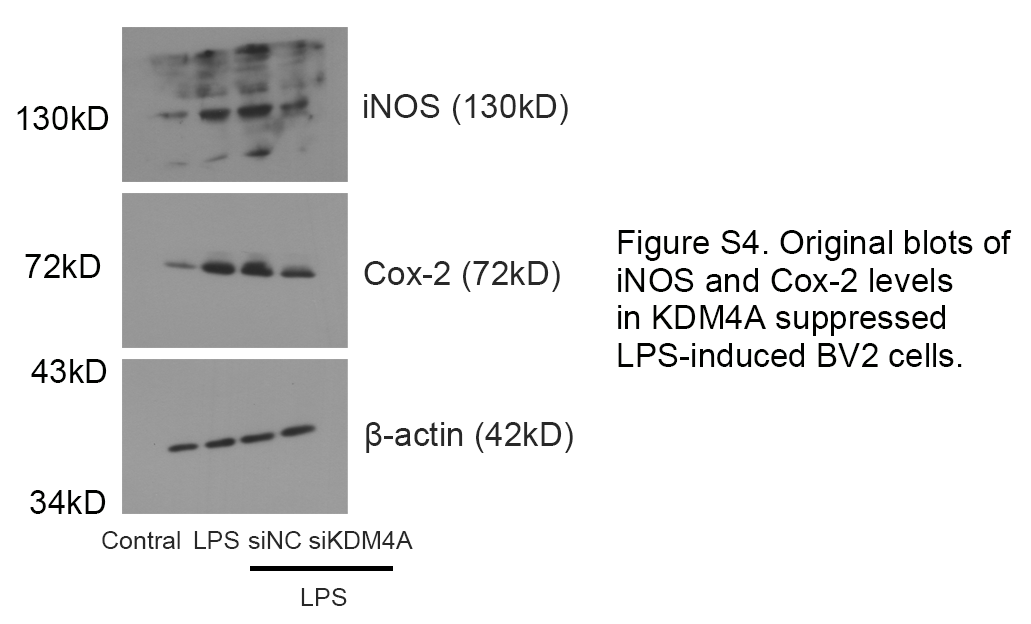

Supplement: Supplementary file 1 — Additional file 1 [file 12868_2023_784_MOESM1_ESM.docx]
